# New Arrangement

**Published:** 1871-10

**Authors:** 


### Editorial and Miscellaneous.

### NEW ARRANGEMENT.

In accordance with a statement in the last number of the Atlanta Medical and Surgical Journal, we now announce that with this number commences an entire change in its management; and, without going into unnecessary details, we assure our readers that it is the determination to make it a permanent publication, not dependent upon the contingency of an enlarged subscription list, while we have the strongest confidence, at the same time, that its circulation will be largely and profitably increased.

As stated upon a former occasion (when the idea of the present more permanent and satisfactory arrangement was conceived), it was perceived that a most favorable opportunity offered for the successful establishment of a Medical Journal of the highest character, of which it was proposed to take advantage by freeing it of the possibility of being regarded in any sense as the organ of any local influence whatever, and by obtaining a regular corps of contributors composed of the most prominent and able Medical men of the North, South and West, who would thus certainly make it an efficient instrument in the advancement of Medical Science generally, but especially of Southern Medicine, by affording a permanent and attractive channel through which an immense amount of valuable but wasting practical experience and knowledge, in our own section, might be rescued for individual and general use. To this end, all individual or other local considerations have been merged into a new arrangement, embracing its publication, its Editorial department, and last, though not least, the organization of a corps of *regular* and *special* contributors, which will, we think very certainly, place it in the front rank of American Medical publications, and secure it a general patronage throughout the Cotton States, and, we hope and believe, to a considerable extent, over the entire country.

No hope of the greater success of the ATLANTA MEDICAL AND SURGICAL JOURNAL (which has already a highly respectable reputation and circulation) upon a higher basis is in any degree indulged, as connected with other journals, in any other sense than as a stimulant to excellence upon the part of all. We *know* that there is an ample field for the abundant success of such a journal as we propose to publish, in even a business point of view, without in any degree interfering with others.

It has been regarded as essential to the advanced position which it was designed for the JOURNAL to occupy *immediately*, that a reliable corps of contributors of the highest character and ability should be secured from the various parts of the United States, which, besides making its original department independent of the contingencies connected with a reliance upon fugitive and irregular communications from a single section, would, also, furnish a medium of communion and interchange of views between the Medical men throughout the widely-extended borders of a common country. Indeed, the positive assurance of success, upon the part of those who have assumed the Editorial responsibility of the JOURNAL, is based more upon the fact that we have been able to combine such an array of talent and scientific attainment among those who have authorized us to promise contributions from their pens, rather than upon any special fitness for such a work upon their own part.

Among this list will be found representatives of Medical Schools and Hospitals, and a large array of the highest Medical and Surgical talent and attainment in New York, Philadelphia, Baltimore, Cincinnati, Louisville, Nashville, New Orleans, Knoxville, Tenn., Columbia, S. C., Mobile and Montgomery, Ala.—besides, in our own State, able representatives of Augusta, Athens, Macon, Columbus, Griffin, Rome, Barnesville, Newnan, West Point, LaGrange, Oxford, and Atlanta—to which we shall be constantly adding, in town or country,

Medical men of ability, who will not be mere figure-heads, but who will promise to aid and sustain us by their labors for our pages.

The Journal will be strictly and absolutely devoted to the highest order of Medical Progress, and will advocate and sustain the most advanced practicable views in regard to Medical Education, and especially a *uniform system throughout the United States*—the result of the combined wisdom of the American Medical Association and the Medical Colleges of the country. It will be devoted exclusively to the highest interests of Medical Science, and will be kept free from the soiling and defacement of every thing *personal*. *No controversy* will be admitted into its pages; and while it will be an arena for Medical discussion, it will be no channel for Medical politics or professional wrangling. It will endeavor not only to promote peace and harmony among the members of a calling whose reputation is not always in accord, in these particlars, with the high objects of so noble a profession, but will endeavor to excite *good will* among all. Our doctrine is, that time is too short and precious to be spent in quarreling, and our own peace of mind is too sacred to allow us to risk its disturbance by countenancing or fostering any contests whatever.

While it has been indispensable to the eminent success of our undertaking, that positive arrangements should be effected for supplying original material of a high order for the pages of the Journal, we yet wish it distinctly understood, that *all* contributions, containing any interesting fact or suggestion connected with the profession, will be acceptable, and will be placed upon precisely the same footing in the order of publication.

